# Effects of Pecan Nut (*Carya illinoiensis*) and Roselle Flower (*Hibiscus sabdariffa*) as Antioxidant and Antimicrobial Agents for Sardines (*Sardina pilchardus*)

**DOI:** 10.3390/molecules24010085

**Published:** 2018-12-27

**Authors:** Juliana Villasante, Marina Girbal, Isidoro Metón, María Pilar Almajano

**Affiliations:** 1 Chemical Engineering Department, Universitat Politècnica de Catalunya, Av.Diagonal 647, 08028 Barcelona, Spain; julianavillasante@gmail.com (J.V.); marinagirbal@gmail.com (M.G.); 2 Department de Bioquímica i Fisiologia, Universitat de Barcelona. Av. de Joan XXIII, 27-31, 08028 Barcelona, Spain; imeton@ub.edu

**Keywords:** pecan nut, roselle flower, *Sardina pilchardus*, health benefits, oxidation, biogenic amines, hexanal

## Abstract

The effects of pecan nut (*Carya illinoinensis*) and roselle flower (*Hibiscus sabdariffa*) as antioxidant and antimicrobial agents on shelf life extension of sardines (*Sardina pilchardus*) were evaluated over a period of 5 days at 7 ± 1 °C. Treatments consisted of the addition of 5% and 10% *w*/*w* pecan nut, 5% *w*/*w* roselle flower and a combination of 5% of each. Physicochemical (lipid oxidation, fatty acids, hexanal and biogenic amines), sensory and microbiological characteristics of fish samples were periodically analyzed. All treatments effectively improved physicochemical quality parameters, with 10% *w*/*w* pecan nut having the highest effectiveness. The presence of roselle flower reduced microbial growth. Our findings suggest that addition of a natural preservative combining pecan nut and roselle flower may extend the shelf life of fresh sardines during chilled storage while maintaining quality indexes.

## 1. Introduction

Sardine (*Sardina pilchardus*) fisheries have an important economic impact on Europe and North Africa. Its production in Europe reached 175 thousand tonnes in 2014, with a value of 161 million euro [1]. Due to its high content of protein and polyunsaturated fatty acids (PUFA) the sardine is a highly perishable fish with remarkable nutritional benefits for human health [2]. Lipid oxidation causes rancidity, off-taste, off-odor and color changes, which can affect consumer’s perception. Synthetic antioxidants such as butylated hydroxyanisole (BHA) and butylated hydroxytoluene (BHT) [3] are used to extend its shelf life, but health problems derived from excessive consumption of these antioxidants have been reported [4]. Therefore, there is increasing concern from consumers and the food industry to avoid the use of artificial antioxidants and find healthier alternatives. The research and use of natural antioxidants present in spices, herbs, fruits, plants or teas containing high levels of phenols, anthocyanins and ascorbic acid (among other compounds, which can act as radical scavengers and prevent the reaction of lipid peroxidation), has increased [5]. Moreover, consumption of plants containing natural phenolic compounds, has been described to prevent free radical formation and reduce the risk of developing cancer, diabetes, cardiovascular disease and Alzheimer’s disease, among others pathologies [6].

Pecan nut (*Carya illinoinensis*) has been shown to contain the highest amount of phytochemicals of all nut types [7]. Studies show strong correlations between inclusion of pecan nut in the diet and reduction of triacylglycerides, LDL cholesterol and increase of HDL colesterol [8]. This beneficial effect in the health comes from the high amounts of phenolics, flavonoids, proanthocyanidins, monounsaturated fatty acids and tocopherols present in pecan nut [9].

Previous research on the use of walnut as an antioxidant for meat showed its effectiveness on preventing food deterioration. In addition, tacking on walnut paste to meat improved MUFA and PUFA contents and amino acid profiles [10].

Roselle (*Hibiscus sabdariffa*) flowers contain high, but variable amounts, depending on the cultivar, of total phenolics, flavonoids and anthocyanidins, and was reported to possess antimicrobial activity for Gram-positive and Gram-negative bacteria [11].

The aim of this research is to test the effectivity of pecan nut as a natural preservative to delay the oxidation of naturally occurring fish lipids. To this effect, sardine was chosen because it is a fish known to contain high amounts of fat, therefore acknowledging that if pecan nut works as a preservative for sardine it could be implied that it would also work for other fish species with less amounts of fat. Also, other objective was to assess the antimicrobial effect of roselle flowers, to be used alone or combined as natural antioxidants or preservatives for the food industry.

## 2. Results and Discussion

### 2.1. Determination of Total Phenolic Content and DPPH Radical Scavenging Activity

The total phenolic content (TPC) of defatted pecan nut and roselle flower were measured on the corresponding ethanol extracts (50%) and no significant difference was found, while the radical scavenging activity (RSA) was significantly higher in defatted pecan kernels than in roselle flower extracts.

TPC content and RSA for defatted pecan nut kernel and roselle flower ethanol extracts varied significantly in previous research. This variability could result from the origin and cultivar of each sample. Table 1 displays the results obtained in this work.

The total phenolic content (TPC) of defatted pecan nut and roselle presented no significant difference (22.95 mg GAE/g FW ± 0.04 and 22.40 mg GAE/g FW ± 0.02), the radical scavenging activity (RSA) is significantly higher in defatted pecan kernels (37.63 mg TE/g FW ± 1.08 and 19.69 mg TE/g FW ± 0.42 for defatted pecan kernels and roselle, respectively). Results expressed as mean ± SD (*n* = 3).

Values for TPC content and RSA for defatted pecan nut kernel vary significantly in the literature: Alasalvar and Shahidi [12] reported similar results for TPC in pecan; Villarreal-Lozoya, Lombardini and Cisneros-Zevallos [13] obtained around double TPC and triple RSA mean values. A review on tree nut phytochemicals by Bolling et al. [7] described a smaller value for TPC and higher for RSA. De la Rosa et al. [8] reported similar but lower RSA pecan nut kernel mean values. Mak et al. [14] obtained a two-fold higher TPC and very similar RSA value for roselle flower ethanol extract and Afify and Hassan [15] described much lower TPC and RSA values. Possible reasons for the great variability found in literature values could be related to the origin and cultivar of each sample.

### 2.2. Microbiological Analysis

Presence of colony-forming units was evaluated in the treatments (control, 5% *w*/*w* PN, 10% *w*/*w* PN, 5% *w*/*w* R, 5% *w*/*w* PN + 5% *w*/*w* R, and 0.1% *w*/*w* BHA) with one and three days of incubation after treatment (Table 2). The main goal of this analysis was to assess the antimicrobial properties of roselle flower and pecan nut as well as checking initial contamination of the samples, with a qualitative analysis. The amount of bacteria present in all samples at day one of incubation was less than 10 CFU/g sample. Three days’ post-treatment showed that roselle flower and BHA acted as antimicrobial agents. Our findings are consistent with previous reports in which roselle flower was successfully used to disinfect carrots, tomatoes [16] and Hass avocado [17]. The results of the present study suggest that roselle flower can be used to supplement the antioxidant activity of pecan nut, hence obtaining a food preservative with both antioxidant and antimicrobial properties.

### 2.3. Thiobarbituric Acid Reactive Substances (TBARS)

**Figure 1:**
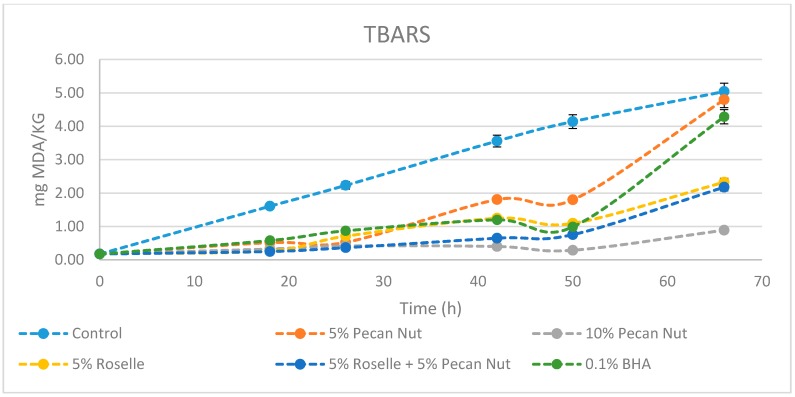
Evolution of TBARS value ^1^ (7 ± 1 °C) in *Sardina pilchardus* samples with different treatments in a period of 66 h. ^1^ Results are expressed in mg MDA/kg.

The TBARS method is widely used for determining the oxidation of fats and oils in foods. Malondialdehyde compounds are formed when the concentration of hydroperoxides is appreciable in oil or fat. Hydroperoxides decompose to form secondary oxidation products [18,19]. Results shown in Figure 1 and Table 3 indicate that after 66 h of incubation in the fridge, samples containing 10% *w*/*w* of pecan nut were significantly less oxidized than in any other condition, including incubation with the artificial antioxidant BHA. Until 50 h all treatments, except the control, follow a similar trend; after, there is a noticeable increase of oxidation rate in samples containing 5% *w*/*w* pecan nut and 0.1% BHA. Samples with 5% *w*/*w* roselle flower and 5% of both pecan nut and roselle flower were also effective in relation to the control. Özogul et al. [20] studied the effects of rosemary and sage tea extracts in preventing lipid oxidation of sardine at 3 °C after six days. Erkan et al. [21] reported TBARS values of sardine storage at 2 °C after five days using thyme and laurel essential oils. Results in both studies and in the present work follow a similar tendency and together support the idea that the addition of natural compounds with antioxidant and antimicrobial activity provides an effective methodology to extend shelf life of fresh sardine.

### 2.4. FA Analysis

FAME analysis was used to assess the amount of FA present in sardine loins and monitor changes among samples containing pecan nut and roselle flower. Results are displayed in Table 4.

FA present in a higher amount in the control and samples treated with 0.1% BHA are myristic acid (C14:0), palmitic acid (C16:0), stearic acid (C18:0), palmitoleic acid (C16:1), oleic acid (C18:1n), erucic acid (C22:1n9), linoleic acid (C18:2n6c), α-linolenic acid (C18:3n3) and *cis*-4,7,10,13,16,19-docosahexaenoic Acid (DHA, C22:6n3). The FA composition ranged from 33.89 to 38.39% of saturated FA (SFA), 23.44 to 27.04% of monounsaturated FA (MUFAs) and 25.04 to 31.89% of polyunsaturated FA (PUFAs). Even though FA amounts in sardine may vary significantly depending on the origin and season, the results of the present study are in agreement with those obtained previously for sardine muscle [22]. FA content in samples incubated with 5% *w*/*w* and 10% *w*/*w* pecan nut vary significantly in relation to the control due to the presence in pecan nuts of amounts four-fold higher of oleic acid (C18:1n) and linoleic acid (C18:2n6c) [23]. As a consequence, total SFA is halved, MUFA doubled and PUFA content remains similar compared to control samples. Therefore, the use of pecan nuts would have a beneficial side effect by increasing the total amount of the healthier types of FA (MUFA and PUFA).

Incubation with 5% *w*/*w* roselle flower did not significantly changed FA amounts compared with control samples. Palmitic acid, oleic acid and DHA are the acids with a higher percentage within control samples. These results are similar to those previous studies [24] reporting the presence of myristic (2.1%), palmitic (35.2%), palmitoleic (2.0%), stearic (3.4%), oleic (34.0%) and linoleic (14.4%) acids in roselle seeds.

All treatments promoted low levels of arachidonic acid (C20:4n6, 0.03–0.58%), which may have antagonistic effects to the health benefits of the n3 FA [25]. Moreover, the UK Department of Health recommends a maximum ratio of n6/n3 of 4.0, which is much higher than in any of the present treatments (0.04–0.07%) [26]. Indeed, a minimum value of PUFA/SFA ratio recommended is 0.45 [26] which is lower than those obtained for all fish treatments (0.65–2.86%).

Palmitic acid was the primary saturated FA, contributing 9.33–26.44% of total SFA in all treatments. Oleic acid was the major MUFA, accounting for 15.03–52.62% of total MUFAs and linoleic acid as well as DHA were the major FA identified as PUFAs, accounting for 6.22–31.30% and 1.04–22.98%, respectively.

### 2.5. Determination of Hexanal by HS-GC-MS

Oxidation of unsaturated fatty acids generates hydroperoxides, highly reactive substances which rapidly decompose into volatile and non-volatile compounds such as hydrocarbons, alcohols, acids, aldehydes and ketones [27]. These are called secondary lipid oxidation products and contribute to flavor and taste deterioration. Among these products, hexanal is a by-product of lipid oxidation that is mainly generated by oxidation of ω-6 fatty acid peroxides, mostly from linoleic acid through 13-hydroperoxide [28].

**Figure 2:**
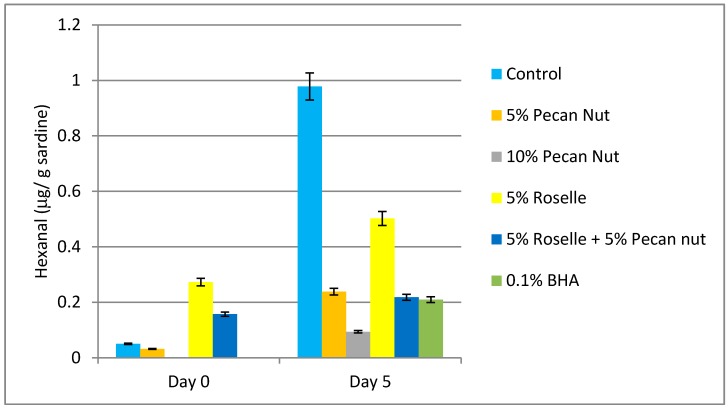
Hexanal content at days 0 and 5 of the experiment in samples of *Sardina pilchardus* with different treatments ^1^. ^1^ Results are expressed in μg hexanal/g sardine. At day 0 the amount of hexanal in the samples with treatments of 10% *w*/*w* pecan nut and 0.1% *w*/*w* BHA are under the limit of detection.

Sample content of hexanal greatly changed during the treatment (Figure 2). At day 5 post-treatment, control samples exhibited the highest levels, which were 20-fold greater than control values at the beginning of the experiment (day 0). The lower levels of hexanal were found in samples containing a 10% *w*/*w* of pecan nut: barely detectable levels at day 0 and slightly increased values at day 5 of incubation. These results suggest that 10% *w*/*w* pecan nut was the most effective treatment for preventing hexanal formation as a by-product of lipid oxidation. Samples with 5% *w*/*w* pecan nut and 0.1% *w*/*w* BHA presented similar increased levels of hexanal after 5 days of treatment, which indicated that 5% *w*/*w* of pecan nut is enough to equal the effects of current artificial antioxidants. Our findings also suggest that roselle flower can prevent hexanal formation.

### 2.6. Biogenic Amine (BA) Analysis

BAs are basic nitrogenous compounds usually generated in foods and beverages by microbial decarboxylation of amino acids or amination and transamination of aldehydes and ketones [29]. In non-fermented foods the presence of BA above a certain level is considered to be indicative of undesired microbial activity. Therefore, the amine level could be used as an indicator of microbial spoilage [29]. The best-known type of food poisoning caused by BA derives from consumption of high levels of histamine. It is also referred to as “scromboid fish poisoning” because of the frequent association of this illness with consumption of scrombroid fish such as tuna, mackerel, saury, bonito, seer fish and butterfly kingfish. Non-scrombroid fish like sardine, anchovy, marline or herring have also been implicated in cases of histamine poisoning [30]. Putrescine and cadaverine, which are present in high levels in toxic fish, have been reported to potentiate the biological effects of histamine up to ten times [31]. In the European Union (EU) the legal limit for histamine levels is 100 mg/kg in raw fish.

In the present study, the amount of BA in sardine flesh significantly varied depending on the treatment (Table 5). Other authors have reported similar results for the quantification of BA in sardine in a period between 3–6 days while stored with refrigeration [20]. As for the effectiveness of the treatments, after 5 days of incubation, samples containing 10% *w*/*w* of pecan nut had much lower amounts of BA than the control and any other treatment. Since BA result from protein decomposition, the results obtained indicate that pecan nut may be more effective in preserving sardine meat than the artificial food preservative BHA. Samples containing 5% *w*/*w* pecan nut showed also better results in preventing the formation of most BA than BHA (in the used concentration). Most BA levels in samples treated with the antimicrobial compound roselle flower were similar or higher than in control samples. Karabacak and Bozkurt [32] reported histamine, putrescine and tyramine concentrations in sucuk batters during the ripening period. In this study, the BA content in samples incubated with roselle flower were similar or smaller than the control, although samples containing roselle flower doubled the amount of tyramine present in controls at day 4, which follows the same tendency shown in this study (Table 5).

### 2.7. Sensory Analysis

In order to know the acceptability of this new product, sensory analysis was performed, using Basker’s tables, establishing a significant difference for 28.5 points. Given that no such difference was found for any of the fish patties included in the preference sensory analysis, no treatment could be established as the preferred one by participants. The results for 5% pecan nut + 5% roselle, 5% pecan nut, control, 10% pecan nut were a total rank of 98, 91, 99 and 80, respectively. General assessor’s comments point out that patties with fish incubated with roselle flower had an acid shade and boosted fish taste. The other three treatments were considered very similar, although pecan nut seemed to soften fishy flavor and even make the sample taste like meat. In fact, lower scores are considered to indicate higher taster’s acceptability and the lowest value was attributed to samples with 10% *w*/*w* pecan nut.

## 3. Materials and Methods

### 3.1. Natural Products

Pecan nuts and roselle flowers purchased in a local market in Mexico were frozen with liquid nitrogen and shredded with a mortar.

### 3.2. Preparation of Extracts for Determination of Total Phenolic Content and DPPH Radical Activity Scavenging

Extracts were prepared in order to assay radical scavenging activity and total phenolic content. Defatted pecan nut kernels and roselle flowers were weighed (1 g) and extracted with 20 mL of 50:50 (*v*/*v*) ethanol-water at and 10 mL of 70:30 (*v*/*v*) ethanol-water with 0.1% (*v*/*v*) HCl 37%, respectively. Pecan nut extract was stirred for 90 min at room temperature and roselle flower extract at 60 °C. Both extracts were centrifuged and the supernatants were stored at −20 °C in darkness until analysis.

### 3.3. Antioxidant Analyses of Extracts of Pecan Nut and Roselle

#### 3.3.1. DPPH Radical Scavenging Activity

Radical scavenging potential of pecan nut and roselle was evaluated using the DPPH method described by Gallego et al. [33]. Results are expressed in μmol Trolox Equivalents (TE)/g of sample weight (SW) ± SD. Measurements were done in triplicate for each sample.

#### 3.3.2. Determination of Total Phenolic Content

The Folin-Ciocalteu (Folin) method was used to measure the total polyphenol content of the extract [34]. Measurements were done in triplicate for each sample. Results are expressed as mg Gallic Acid Equivalents (GAE)/g of SW ± SD.

### 3.4. Fish Sample Preparation

Fresh sardines with an average weight and length of 27.2 g ± 7.5 and 14.8 cm ± 1.25 respectively, results expressed as mean ± SD (standard deviation) and with *n* = 10, were purchased from a local market in Barcelona, and transported under refrigeration to the laboratory. Fish were gutted and head, tail and spine were removed, keeping only the loins.

### 3.5. Preparation of Samples

Sardine patties were prepared in order to measure the evolution of a range of parameters throughout a period of time. Burgers were prepared by shredding sardine flesh and adding direct and minced pecan nut and rosolle with the following treatments: control (1% *w*/*w* salt), 5% PN (5% *w*/*w* pecan nut, 1% salt), 10% PN (10% *w*/*w* pecan nut, 1% salt), 5%R (5% *w*/*w* roselle, 1% salt), 5%PN + 5%R (5% *w*/*w* pecan nut, 5% *w*/*w* roselle, 1% salt), 0.1% BHA (0.1% *w*/*w* BHA, 1% salt). Samples were kept at 7 ± 1 °C until analysis.

### 3.6. Microbiological Analysis

Microbiological analysis was performed to assay sample contamination with mesophilic bacteria. To perform the analysis, 10 g of sample were added to 90 mL of Ringer solution and homogenized with a Stomacher for 5 min. Triptone Soya Agar (TSA) was used as the growth media and plaques were incubated at 35 °C. The recount was performed after 24 h and 48 h.

### 3.7. Thiobarbituric Acid Reactive Substances (TBARS)

Determination of TBARS value was performed following the method described by Gallego et al. [34] with some modification. Triplicates of 0.5 g for each sample were weighed, added 0.5 mL 0.3% EDTA solution and 2.5 mL TBARS reagent and homogenized with an Ultra-Turrax blender (Ika-Werke, GmbH & Co, Staufen, Germany) for 1 min. During all procedure’s tubes were kept in an ice bath to prevent sample deterioration. They were then filtered with Whatman filters no. 1 and the reaction was activated through insertion of the tubes in a water bath at 95 ± 1 °C for 10 min. After cooling RT, absorbance was measured at 531 nm in a UV/VIS microplate reader spectrophotometer Fluostar Ω (Paris, France). Results were expressed as mg of malondialdehyde (MDA)/kg sample.

### 3.8. Fatty Acid Methyl Ester (FAME) Analysis

Fatty acids (FA) analysis was performed according to the method described by Viegas et al. [35] with modifications. Duplicates of 200 mg of sample were weighed into glass tubes and 750 µL of methanol:water solution 2:1 (*v*/*v*), followed by 500 µL of chloroform and 250 µL Milli-Q water were added, vortexing for 1 min after each addition. Thereafter, the samples were centrifuged at 2000 g, 4 °C for 20 min. The lower layer was transferred to opaque vials and evaporated at 25 °C, with a nitrogen stream until only oil residue was present. Following addition of 2 mL hexane, the samples were vortexed for 30 s and left to rest for 5 min, to ensure fat dilution in hexane. Afterwards, 200 mL 2 M potassium hydroxide in methanol solution were added and the samples centrifuged for 10 min at 2000 g. The upper phase was transferred into a 2 mL tube and kept at −80 °C until gas chromatography analysis was performed.

FA composition was analyzed using a GC-2025 with autosampler (Shimadzu, Tokyo, Japan), equipped with a flame ionization detector (FID) and a BPX-70 (SGE) column (L × I.D. 30 m × 0.25 mm, d_f_ 0.25 μm). Temperature in the oven was 60 °C for 1 min and then it was raised to 260 °C at the rate of 6 °C/min, while the injector and the detector temperatures were set at 260 and 280 °C, respectively. Sample volume was 1 mL and the carrier gas was helium. The split used was 1:20. FAME were identified by comparing the retention times of standard 37 component FAME mixture. Two replicate GC analyses were performed and the results were expressed in GC area % as mean values ± sd.

### 3.9. Determination of Hexanal by HS-GC/MS

0.5 g of minced sample was added to 1.5 mL milli-Q water in a headspace vial, which was then sealed air-tight with a PTFE septum. The standard curve was prepared using hexanal with concentrations ranging from 0.005 to 0.450 ppm. Results were expressed in mg hexanal/g sardine.

The vials were incubated at 80 °C during 30 min. The analysis was performed by HS-GC/MS, by injecting 1 mL of vapor phase through a special syringe kept at 85 °C. Equipment used consisted of a Trace GC gas chromatograph with a Head Space Triplus autosampler coupled to a DSQII mass spectrometer (Thermo Fisher Scientific, Austin, Texas, USA) with TRB-624 (60 m × 0.32 mm × 1.8 mm) column, 1.8 mL/min helium flow. The injector temperature was 220 °C with split mode injection (split flow 20 mL/min). Temperature program was 60 °C held for 2 min and then raised to 220 °C at the rate of 8 °C/min (5 min). Interface temperature was 260 °C and ionization source temperature 230 °C. Ionization mode: electron ionization, SCAN mode (29–250 amu).

### 3.10. Biogenic amines (BA) Analysis

Samples were prepared according to the method of Komprda et al. [36] with some modifications. 1 g of sample was extracted with 2 mL of HCl 0.1 M and homogenized using an Ultra-Turrax blender (Ika-Werke, GmbH & Co, Staufen, Germany) for 1 min. Homogenized samples were centrifuged for 15 min at 4 °C and 2000 g. The supernatant was separated and the solid residue was repeatedly extracted with 2 mL of HCl 0.01 M, vortexed for 30 s and centrifuged for 15 min with the same conditions. The supernatant was separated again and the combined extracts were made up to 10 mL. The samples were filtered through 0.45 µm filter prior to liquid chromatography analysis.

BA analysis was performed following the method of Hernández-Jover et al. [37]. As biogenic amine standards histamine (HIS), tyramine (TYR), serotonin (SER), tryptamine (TRP), octopamine (OC) hydrochloride, dopamine (DO) 3-hydroxytyramine hydrochloride, cadaverine (CAD), putrescine (PUT), spermine (SPM), and spermidine (SPD) were used. A concentrated 1000 mg/L stock solution as a free base for each biogenic amine in 0.1 M HCl was prepared. A 50 mg/L intermediate solution was prepared in 0.1 M HCl from the stock solution. Calibration standards of 0.25 mg/L for all amines and 2 mg/L for spermine were prepared in 0.1 M HCl from the intermediate standard solution, stored at 4 °C and protected from light. The HPLC analysis parameters, mobile phase, gradient program, postcolumn derivatizating reagent and other procedures were as described in Hernández-Jover et al. [37].

### 3.11. Preference Sensory Analysis

Sensory analysis was conducted by a taste panel consisting of 37 semi-trained judges (21 males and 16 females) with age ranging from 17 to 60. All participants declared that they do not suffer from dried fruits allergy. Participants tasted four fish patties, each corresponding to 18 h of incubation of sardine loins with the following additions: 5% *w*/*w* pecan nut, 10% *w*/*w* pecan nut, 5% *w*/*w* pecan nut + 5% *w*/*w* roselle flower and control (nothing added). The samples were distributed in plates and coded with a random three digit number. The subjects were instructed to taste each sample and grade them from 1 (most preferred) to 4 (least preferred). Results were analyzed using the tables developed by Basker [38].

### 3.12. Statistical Analysis

The mean value and standard deviation were calculated from the data obtained from the three samples for each treatment. Where significant differences were detected by one-way Anova, means were compared using Turkey’s test *p* < 0.05. All statistics were performed using Minitab-16 for Windows software (Pennsylvania State University, State College, PA, USA).

## 4. Conclusions

The study showed those pecan nut and roselle flowers are highly effectivity as a fish preservative, thus opening the way to perform other experiments varying the fish type or even switching to pecan nut byproducts such as leaves or shell. It can be understood as a preliminary study to assess the interaction between pecan nut and fish lipids. All analyses showed that samples treated with pecan nut and roselle flower had better quality parameters than the control.

Adding pecan nut to sardine, creates a functional product which is useful. First, because it enhances the shelf-life of sardine and second because it has an increased percentage of healthy monounsaturated fatty acids and a smaller percentage of saturated fatty acids. This research shows that when adding pecan nut to sardine the amount of linoleic acid (Ω-6 fatty acid family) is more than tripled (from an average percentage of 15% to 50% of the total amount of fatty acids). Linoleic acid is one of the two essential fatty acids that need to be ingested through food since the body cannot synthesize them. The other most notable change when adding pecan nut to sardine is the increase of the average percentage of oleic acid (Ω-9 family) from 6.5% to 28% (more than four times greater). This monounsaturated fatty acid is also known for its beneficial effects on health such as reducing blood pressure. Pecan nut alone or in combination with roselle flower has potential to be used as a natural food preservative for the food industry.

## Figures and Tables

**Table 1:** Total phenolic compounds and radical scavenging activity ^1^.

Sample	TPC (mg GAE/g FW)	RSA (mg TE/g DW)
Defatted Pecan Nut kernel	22.95 ± 0.04	37.63 ± 1.08
Roselle flower	22.40 ± 0.02	19.69 ± 0.42

^1^ Results were expressed as miligrams gallic acid equivalents (GAE) per gram of fresh sample weight (FW) and miligrams of Trolox equivalents (TE) per gram of dry sample.

**Table 2:** Presence of mesophilic bacteria in the samples ^1^.

Treatment	Day 1	Day 3
Control	-	+
5%Pecan Nut	-	+
10% Pecan Nut	-	+
5% Roselle	-	-
5% Roselle + 5%Pecan Nut	-	-
0.1% BHA	-	-

^1^ indicates there is no presence; + an amount between 30 and 100 CFU/g.

**Table 3:** Evolution of TBARS values (mg MDA/kg) ^1^ in *Sardina pilchardus* samples at 18, 50 and 66 h of incubation at 7 ± 1 °C.

Treatment	Hours		
	18	50	66
Control	1.61 ^a^ ± 0.05	4.14 ^a^ ± 0.25	5.04 ^a^ ± 0.01
5% Pecan Nut	0.52 ^bc^ ± 0.13	1.80 ^b^ ± 0.35	4.80 ^a^ ± 0.01
10% Pecan Nut	0.33 ^cd^ ± 0.03	0.29 ^d^ ± 0.01	0.89 ^c^ ± 0.15
5% Roselle	0.27 ^d^ ± 0.02	1.10 ^c^ ± 0.08	2.33 ^b^ ± 0.23
5% Roselle + 5% Pecan Nut	0.25 ^d^ ± 0.02	0.76 ^cd^ ± 0.03	2.18 ^b^ ± 0.43
0.1% BHA	0.58 ^b^ ± 0.06	0.99 ^b^ ± 0.08	4.29 ^a^ ± 0.02

^1^ Results are expressed as mean ± SD (*n* = 3). ^a,b,c,d^ Different letters in the same column indicate significant differences (*p* < 0.05).

**Table 4:** Fatty acids profiles of *Sardina pilchardus* muscle with different treatments ^1.^

Fatty Acids (%)	Control	5% Pecan Nut	10% Pecan Nut	5% Roselle	5% Roselle + 5% Pecan Nut	0.1% BHA
C6:0	0.00 ± 0.00	0.00 ± 0.00	0.00 ± 0.00	0.00 ± 0.00	0.00 ± 0.00	0.00 ± 0.00
C8:0	0.20 ± 0.28	0.02 ± 0.02	0.01 ± 0.01	0.17 ± 0.23	0.02 ± 0.03	0.00 ± 0.00
C10:0	0.18 ± 0.26	0.02 ± 0.02	0.02 ± 0.02	0.13 ± 0.19	0.02 ± 0.03	0.06 ± 0.09
C11:0	0.00 ± 0.00	0.00 ± 0.00	0.00 ± 0.00	0.00 ± 0.00	0.00 ± 0.00	0.00 ± 0.00
C12:0	0.00 ± 0.00	0.01 ± 0.02	0.01 ± 0.01	0.00 ± 0.00	0.02 ± 0.02	0.06 ± 0.08
C13:0	0.00 ± 0.00	0.00 ± 0.00	0.00 ± 0.00	0.00 ± 0.00	0.00 ± 0.00	0.00 ± 0.00
C14:0	2.54 ^a^ ± 0.10	0.63 ^b^ ± 0.14	0.29 ^b^ ± 0.06	2.49 ^a^ ± 0.24	0.71 ^b^ ± 0.05	3.22 ^a^ ± 0.78
C15:0	0.54 ± 0.01	0.12 ± 0.02	0.06 ± 0.01	0.54 ± 0.06	0.14 ± 0.01	0.72 ± 0.15
C16:0	24.29 ^a^ ± 0.71	11.40 ^bc^ ± 0.76	9.33 ^c^ ± 0.59	24.75 ^a^ ± 0.71	12.04 ^b^ ± 0.38	26.01 ^a^ ± 0.58
C17:0	0.45 ± 0.03	0.14 ± 0.01	0.10 ± 0.00	0.47 ± 0.05	0.17 ± 0.00	0.64 ± 0.20
C18:0	3.82 ^a^ ± 0.19	2.52 ^b^ ± 0.08	2.35 ^b^ ± 0.07	3.35 ^a^ ± 0.19	2.54 ^b^ ± 0.05	3.95 ^a^ ± 0.38
C20:0	0.82 ± 0.06	0.14 ± 0.04	0.07 ± 0.00	0.20 ± 0.10	0.19 ± 0.01	0.57 ± 0.81
C21:0	0.07 ± 0.10	0.01 ± 0.02	0.01 ± 0.01	0.12 ± 0.01	0.01 ± 0.02	0.19 ± 0.04
C22:0	0.67 ± 0.04	0.13 ± 0.05	0.05 ± 0.01	0.68 ± 0.11	0.16 ± 0.00	0.83 ± 0.23
C23:0	0.00 ± 0.00	0.00 ± 0.00	0.00 ± 0.00	0.00 ± 0.00	0.00 ± 0.00	0.00 ± 0.00
C24:0	0.69 ± 0.10	0.10 ± 0.01	0.04 ± 0.04	0.80 ± 0.15	0.16 ± 0.04	0.67 ± 0.01
ƩSFA ^2^	33.89	15.19	12.30	33.40	16.13	36.87
C14:1	0.00 ± 0.00	0.01 ± 0.02	0.00 ± 0.00	0.05 ± 0.08	0.01 ± 0.02	0.08 ± 0.11
C15:1	0.07 ± 0.10	0.01 ± 0.02	0.00 ± 0.00	0.06 ± 0.08	0.01 ± 0.02	0.07 ± 0.10
C16:1	1.72 ^a^ ± 0.04	0.39 ^b^ ± 0.09	0.20 ^b^ ± 0.03	1.65 ^a^ ± 0.19	0.49 ^b^ ± 0.01	2.12 ^a^ ± 0.42
C17:1	0.18 ± 0.06	0.05 ± 0.04	0.07 ± 0.01	0.14 ± 0.04	0.03 ± 0.00	0.10 ± 0.14
C18:1n	15.03 ^b^ ± 1.19	48.49 ^a^ ± 2.02	52.62 ^a^ ± 1.83	16.24 ^b^ ± 0.35	46.1 ^a^ ± 1.37	15.62 ^b^ ± 3.44
C20:1n9	0.23 ± 0.10	0.22 ± 0.00	0.21 ± 0.02	0.45 ± 0.41	0.22 ± 0.01	0.67 ± 0.21
C22:1n9	5.76 ^a^ ± 0.06	0.96 ^b^ ± 0.20	0.46 ^b^ ± 0.12	5.70 ^a^ ± 0.33	1.34 ^b^ ± 0.04	6.39 ^a^ ± 0.53
C24:1	0.46 ± 0.23	0.09 ± 0.06	0.03 ± 0.05	0.54 ± 0.25	0.11 ± 0.05	0.56 ± 0.37
ƩMUFA ^2^	23.44	50.23	53.60	24.82	48.32	25.61
C18:2n6t	0.10 ± 0.14	0.00 ± 0.00	0.01 ± 0.01	0.00 ± 0.00	0.01 ± 0.01	0.12 ± 0.04
C18:2n6c	6.57 ^b^ ± 0.56	28.15 ^a^ ± 0.40	29.32 ^a^ ± 0.70	7.25 ^b^ ± 0.10	27.55 ^a^ ± 0.57	6.22 ^b^ ± 2.18
C18:3n3	0.17 ^a^ ± 0.02	0.67 ^a^ ± 0.92	1.19 ^a^ ± 0.02	0.39 ^a^ ± 0.39	1.27 ^a^ ± 0.02	0.53 ^a^ ± 0.41
C18:3n6	0.64 ± 0.02	0.66 ± 0.78	0.10 ± 0.01	0.71 ± 0.02	0.11 ± 0.00	0.44 ± 0.39
C20:2	0.00 ± 0.00	0.00 ± 0.00	0.00 ± 0.00	0.00 ± 0.00	0.00 ± 0.00	0.00 ± 0.00
C20:3n6	0.31 ± 0.26	0.05 ± 0.08	0.00 ± 0.00	0.06 ± 0.08	0.00 ± 0.00	0.07 ± 0.10
C20:4n6	0.24 ± 0.34	0.04 ± 0.06	0.04 ± 0.01	0.58 ± 0.15	0.12 ± 0.01	0.51 ± 0.00
C20:3n6	0.00 ± 0.00	0.01 ± 0.02	0.01 ± 0.01	0.00 ± 0.00	0.00 ± 0.00	0.00 ± 0.00
C20:5n3	0.87 ± 0.45	0.13 ± 0.08	0.07 ± 0.03	0.51 ± 0.17	0.09 ± 0.02	0.58 ± 0.49
C22:2	0.00 ± 0.00	0.00 ± 0.00	0.00 ± 0.00	0.00 ± 0.00	0.00 ± 0.00	0.00 ± 0.00
C22:6n3	22.98 ^a^ ± 0.27	3.59 ^bc^ ± 0.35	1.75 ^c^ ± 0.57	23.79 ^a^ ± 1.11	4.87 ^b^ ± 0.44	22.68 ^a^ ± 0.31
ƩPUFA ^2^	31.89	33.29	32.50	33.28	34.01	31.15
PUFA/SFA ^3^	0.94	2.19	2.64	1.00	2.11	0.84
Ʃn6	1.29	0.76	0.16	1.35	0.24	1.14
Ʃn3	24.03	4.39	3.02	24.68	6.22	23.78
n6/n3 ^4^	0.05	0.17	0.05	0.05	0.04	0.05
DHA/EPA ^5^	26.38	27.60	25.08	47.02	55.97	39.33
Unidentified	10.40	1.26	1.58	8.20	1.50	6.31

^1^ Results expressed as percentage of total FAME. The values are means ± S.D. of the samples analyzed in duplicate. ^a,b,c^ The means followed by different letters in the same row indicate significant differences (*p* < 0.05). ^2^ Saturated, monounsaturated and polyunsaturated fatty acids. ^3^ Ratio of polyunsaturated to saturated fatty acids. ^4^ Ratio of Ʃn6 to Ʃn3. ^5^ Ratio of *cis*-4,7,10,13,16,19-docosahexaenoic acid (DHA, C22:6n3) to *cis*-5,8,11,14,17-eicosapentaenoic acid (EPA, C20:5n3).

**Table 5:** Biogenic amines present in *Sardina pilchardus* meat samples with different treatments at day 5 ^1^.

	Control	5% Pecan Nut	10% Pecan Nut	5% Roselle	5% Roselle + 5% Pecan Nut	0.1% BHA
OC	8.02 ^a^ ± 7.83	5.93 ^a^ ± 1.91	5.94 ^a^ ± 3.68	4.48 ^a^ ± 4.57	9.12 ^a^ ± 7.59	6.76 ^a^ ± 8.74
DO	4.73 ^a^± 3.10	0.68 ^a^ ± 0.50	1.36 ^a^ ± 0.00	8.27 ^a^ ± 0.98	10.26 ^a^ ± 3.62	7.57 ^a^ ± 6.25
PUT	3.34 ^b^ ± 0.83	3.19 ^b^ ± 1.86	1.26 ^b^ ± 0.36	22.76 ^a^ ± 5.10	19.92 ^a^ ± 0.20	5.97 ^b^ ± 4.00
TYR	4.61 ^a,b,c^ ± 0.76	4.97 ^a,b^ ± 0.88	1.14 ^c^ ± 0.55	7.92 ^a^ ± 1.24	8.24 ^a^ ± 0.39	2.13 ^b,c^ ± 1.30
CAD	1.03 ^a^ ± 0.20	0.78 ^a^ ± 0.18	0.10 ^a^ ± 0.03	0.99 ^a^ ± 0.76	1.08 ^a^ ± 0.73	0.81 ^a^ ± 0.37
SER	11.80 ^a^ ± 4.45	12.41 ^a^ ± 1.57	4.51 ^a^ ± 3.33	30.39 ^a^ ± 5.71	21.91 ^a^ ± 17.55	15.78 ^a^ ± 2.08
HIS	1.62 ^b^ ± 1.11	1.53 ^b^ ± 0.12	0.90 ^b^ ± 0.58	14.08 ^a^ ± 1.23	11.69 ^a^ ± 2.88	4.49 ^b^
SPD	7.28 ^a^	6.14 ^a^	6.55 ^a^ ± 2.47	ND	ND	7.17 ^a^
TRP	6.64 ^a^	5.42 ^a^	2.01 ^a^ ± 1.62	ND	ND	10.34 ^a^
SPM	ND	ND	6.48	ND	ND	ND

^1^ Results are expressed as mean ± standard deviation in mg/100 g sardine. ND = not detected. ^a,b,c^ The means followed by different letters in the same row indicate significant differences (*p* < 0.05) on amine levels in treatments.

## Abbreviations

TPC	Total Polyphenol Content
ABTS	2,2′azino-bis(3-ethyl-benzothiazoline-6-sulfonicacid)
Trolox	(±)-6-hydroxy-2,5,7,8-tetramethylchromane-2-carboxylic acid
GAE	mg of gallic acid equivalents
DW	dry weight
HPLC	High Performance Liquid Chromatography
TEAC	Trolox Equivalent Antioxidant Capacity
TE	Trolox Equivalent
TBARs	Thiobarbituric Acid Reactive substances
BHA	Butylated hydroxyanisole
BHT	Butylated hydroxytoluene
r.t.	room temperature
AMU	atomic mass unit
BA	Biogenic amines
CAD	Cadaverine
CFU	Colony-forming units
DHA	*cis*-4,7,10,13,16,19-docosahexaenoic acid
DO	Dopamine
DPPH	2,2-diphenyl-1-picrylhydrazyl
EDTA	Ethylenediaminetetraacetic acid
EPA	*cis*-5,8,11,14,17-eicosapentaenoic acid
EU	European Union
FA	Fatty Acids
FAME	Fatty Acids Methyl Ester
FID	flame ionization detector
Folin	Folin-Ciocalteu Analysis
FRAP	Ferric reducing antioxidant power
FW	Fresh weight
GC	Gas chromatography
HIS	Histamine
HS-GC/MS	Headspace gas chromatography mass spectrometry
MDA	Malonaldehyde
MUFA	monounsaturated fatty acids
n3	n3-polyunsaturated fatty acid
n6	n6-polyunsaturated fatty acid
OC	Octopamine
OPT	o-Ophtalaldehyde
PN	Pecan Nut
PTFE	Polytetrafluoroethylene
PUFA	polyunsaturated fatty acids
PUT	putrescine
RSA	Radical scavenging activity
SD	Standard deviation
SER	Serotonine
SFA	Saturated fatty acids
SPD	Spermidine
SPM	Spermine
SW	Sample weight
TRP	Ttryptamine
TSA	Tryptone soya agar
TYR	Tyramine
UV/VIS	Ultraviolet-visible spectroscopy
*v*/*v*	volume volume
*w*/*w*	weight weight
RT	Room Temperature
